# The Different Effects of IFN-*β* and IFN-*γ* on the Tumor-Suppressive Activity of Human Amniotic Fluid-Derived Mesenchymal Stem Cells

**DOI:** 10.1155/2019/4592701

**Published:** 2019-04-15

**Authors:** Jingchun Du, Anqi Liu, Rui Zhu, Chengbo Zhou, Heng Su, Guie Xie, Yingshan Deng, Xia Xu

**Affiliations:** ^1^Department of Clinical Immunology, Kingmed School of Laboratory Medicine, Guangzhou Medical University, Guangzhou, Guangdong 510182, China; ^2^Department of Obstetrics and Gynecology, The First Affiliated Hospital of Guangzhou Medical University, Guangzhou, Guangdong 510120, China

## Abstract

Current studies have shown that type I or II interferon-modified mesenchymal stem cells have great potential for the application of tumor-targeted therapy, but the underlying mechanism remains largely elusive. Here, we compared the different effects of IFN-*β* and IFN-*γ* on the antitumor activity of human amniotic fluid-derived mesenchymal stem cells (AFMSCs) and revealed the potential mechanism. In detail, AFMSCs primed with IFN-*β* or IFN-*β* plus IFN-*γ*, not IFN-*γ*, inhibited the proliferation of cancer cells in an immunocompetent mouse H460 subcutaneous model, although they all inhibited the proliferation of cancer cells in an immunocompromised mouse H460 subcutaneous model. TRAIL expressed by IFN-*β*- or IFN-*γ*-primed AFMSCs specifically exerted the antitumor effect of AFMSCs. AFMSCs primed with IFN-*γ* highly expressed immunosuppressive molecule IDO1, but IFN-*β* counteracted the IFN-*γ*-initiated IDO1 expression. 1-MT (IDO1 inhibitor) decreased TRAIL, but increased IDO1 expression in AFMSCs primed with interferon. As a result, AFMSCs primed with IFN-*β* or IFN-*γ* had the antitumor activity, and 1-MT failed to enhance the antitumor effect of IFN-*γ*-primed AFMSC *in vitro* and in the immunocompromised mouse H460 subcutaneous model. Furthermore, the expression of TRAIL in AFMSCs was upregulated by apoptotic cancer cells and this positive feedback intensified the antitumor effects of IFNs-primed AFMSCs. The different target gene expression profiles of AFMSCs regulated by IFN-*β* and IFN-*γ* determined the different antitumor effects of IFN-*β*- and IFN-*γ*-primed AFMSCs on tumor cells. Our finding may help to explore a clinical strategy for cancer intervention by understanding the antitumor mechanisms of MSCs and interferon.

## 1. Introduction

Interferons (IFNs) are a family of structurally related cytokines consisting mainly of type I IFN (*α*, *β*, *ε*, *κ*, *ω*, and *δ*) and type II IFN (IFN-*γ* only). After interacting with the type-specific receptor complex, IFNs trigger the JAK-STAT pathway together with additional signaling cascades [1]. In addition to controlling viral infection, IFNs also exhibit antitumor activity and immunoregulatory effects [2]. Besides immune cells (DCs, T, and NK cells), nonimmune cells might also participate in IFN-mediated antitumor activity. For example, IFN-*β* could exert antitumor activity via signals on nonhematopoietic Tie2^+^ cells, which is associated with extensive inhibition of angiogenesis [3, 4]. However, IFN-related cell targets and antitumor mechanism are not fully recognized because of the almost ubiquitous expression of IFN receptors and the pleiotropic effects initiated by IFNs and their specific receptor interaction.

Mesenchymal stem cells (MSCs) are considered to be appropriate gene carriers for tumor therapy on account of their tropism to tumor tissue, which can be isolated and cultured from many tissues (e.g., bone marrow, adipose tissue, and placenta) [5, 6]. In recent years, amniotic fluid has been recognized as an alternative source of MSCs because it could be collected safely during amniocentesis in the second and third trimesters and there are few ethical concerns associated with its isolation [7]. However, the safety of MSCs remains in question, as current opinions on the role of MSCs in cancer development are contradictory. For example, some reports have shown that MSCs promoted tumor growth, but others disagreed [8–10]. The cytokines around MSCs may be an important factor leading to different effects of MSCs on tumor progression [11, 12]. Our previous studies have also shown that IFN-*γ* could initiate the antitumor activity of MSCs, although this effect was not satisfactory *in vivo* [13].

Based on these data, it could be found that type I and II IFNs affect the antitumor activity of MSCs. But how IFNs affect the antitumor activity of MSCs in detail and whether there exist differences between type I and type II IFNs in spurring the antitumor activity of MSCs are unclear and deserve further exploration.

## 2. Materials and Methods

### 2.1. Cell Cultures

The two-stage culture protocol for isolating MSCs from amniotic fluid were performed, as previously described with some modifications [14]. Briefly, the amniotic fluid samples (20 ml) were obtained from 16-20-week pregnant women who had given informed consent and underwent amniocentesis for prenatal diagnosis. The sample was centrifuged at 2000 rpm for 10 minutes, the supernatant was removed, and the cell pellet was resuspended in 5 ml of growth medium BIOAMFTM-2 (BI, Beit Haemek, Israel) and plated in a 25 cm^2^ culture flask (Corning, Lowell, NY). The first-stage amniocytes were incubated for 7-15 days at 37°C in 5% CO_2_ and then used for fetal karyotyping. For culturing AFMSCs, the nonadherent amniotic fluid cells were collected from primary culture and further expanded in a new 25 cm^2^ culture flask with low-glucose DMEM supplemented with 10% (*v*/*v*) fetal bovine serum (FBS), 100 U/ml penicillin, 100 *μ*g/ml streptomycin (Gibco, Grand Island, NY), and 2 ng/ml bFGF (PeproTech, Rocky Hill, NJ) at 37°C in 5% CO_2_ and 8% O_2_. All AFMSCs were cultured in this condition before they were cocultured with tumor cells or used in animal experiments. The AFMSCs were subcultured into higher passages at about 90% confluence with 0.25% trypsin-EDTA (Gibco, Grand Island, NY). The AFMSCs at passages 5-12 were derived from at least 5 samples and used in all experiments.

Human H460 cells were obtained from ATCC and cultured in RPMI 1640 media supplemented with 10% (*v*/*v*) fetal bovine serum (FBS), 100 U/ml penicillin, and 100 *μ*g/ml streptomycin (Gibco, Grand Island, NY). H460 cells were transduced with lentiviral particles carrying the hrGFP gene, and then the cells stably expressing hrGFP, which were termed H460 hrGFP, were selected by puromycin and established as previously described [13]. Media were changed every 2-3 days, and cells were passaged every 2-3 days. All experiments with H460 and H460 hrGFP were performed at no more than 3-5 cell passages.

### 2.2. Identification of AFMSCs

For colony-forming unit-fibroblast (CFU-F) assay, 100 AFMSCs per well were plated in a 6-well plate and incubated at 37°C for 10-14 days in a humidified 5% CO_2_ and 8% O_2_ incubator. The media were changed every 3 days. The plates were washed with PBS, and cells were fixed with 4% (*w*/*v*) paraformaldehyde and then stained with 1% (*w*/*v*) crystal violet (Sigma-Aldrich, St. Louis, MO) at room temperature. After 5 minutes of staining, cells were washed with distilled water. All colonies containing >50 cells were counted. The colony-forming efficiency of AFMSCs was calculated by dividing the number of colonies by the number of cells seeded per well. Three replicate plates were used, and mean values were calculated.

For cell proliferation capacity assay, 100 *μ*l of AFMSC suspension was seeded into a 96-well plate at 1000 cells per well and cultured at 37°C for 7 days in 5% CO_2_ and 8% O_2_. The media were changed every 3 days. Every two days, the number of cells was evaluated using the CCK-8 kit (Dojindo, Kumamoto, Japan). At the same time, another group of AFMSCs was cultured at 37°C in 5% CO_2_ and 21% O_2_ as a control.

For osteogenic and adipogenic differentiations of AFMSCs in vitro, the methods described in our previous report [13] were used.

For analysis of cell surface antigens, AFMSCs were trypsinized, washed with PBS, and incubated with the following antibodies against cell surface markers: fluorescein isothiocyanate-conjugated (anti-CD44, anti-CD45), phycoerythrin-conjugated (anti-CD34, anti-CD73), PerCP-Cy™5.5-conjugated (anti-CD105, HLA-DR), APC-conjugated (anti-HLA-A, B, and C), or PerCP-Cy™7-conjugated (anti-CD90) antibodies. Irrelevant isotype-matched antibodies were used as negative control. All antibodies were from BD Pharmingen (Palo Alto, CA). Flow cytometry analyses were performed with a Beckman CytoFLEX flow cytometer.

For karyotyping analysis, AFMSCs at passages 1, 16, and 30 were incubated at 37°C with a final concentration of 1 *μ*g/ml colchicine (Sangon Biotech, Shanghai, China) for 2 hours before harvest. The cells were fixed and spread according to standard procedures. The metaphases of AFMSCs were G-banded and karyotyped in accordance with the International System for Human Cytogenetic Nomenclature recommendations (2013).

### 2.3. Quantitative Reverse Transcription-Polymerase Chain Reaction (qRT-PCR)

Total RNAs were extracted using TRIzol reagent (Invitrogen, Carlsbad, CA) after AFMSCs were primed with IFN-*β* or IFN-*γ* (20 ng/ml, PeproTech, USA) for 12 hours in low-glucose DMEM supplemented with 10% (*v*/*v*) FBS. First-strand cDNA synthesis was performed using a reverse transcription PCR kit, according to the manufacturer's instructions (Fermentas, Glen Burnie, MD). The mRNA of interest genes was quantitated by real-time PCR (AriaMx, Santa Clara, CA) using SYBR Green Master Mix (TaKaRa, Beijing, China). The total amount of mRNA was normalized to endogenous *GAPDH* mRNA. Sequences of RT-PCR primer pairs were as follows: TRAIL, forward 5′-CTC TGT GTG GCT GTA ACT TAC GTG-3′ and reverse 5′-CAA GCA ATG CCA CTT TTG GA-3′; IDO1, forward 5′-GCC CTT CAA GTG TTT CAC CAA-3′ and reverse 5′-CCA GCC AGA CAA ATA TAT GCG A-3′; and GAPDH, forward 5′-GAA GGT GAA GGT CGG AGT C-3′ and reverse 5′-GAA GAT GGT GAT GGG ATT TC-3′.

### 2.4. Western Blot Analysis

AFMSCs were washed with cold PBS and lysed with cell lysis buffer (CST, Beverly, MA) after 12 hours of IFN treatments (20 ng/ml each) with or without the Jak1 inhibitor (15 *μ*M) or 1-MT (0.5 mM). The lysates were centrifuged to remove insoluble material. The protein concentration in supernatants was measured with BCA Protein Assay Reagent (Thermo Scientific, Rockford, IL). The same amounts of protein from each group were separated by SDS-PAGE on 12% or 15% gels and then transferred to the polyvinylidene difluoride (PVDF) membranes (Millipore, Billerica, MA). The membranes were blocked with a solution of 0.1% (*v*/*v*) Tween-20/TBS (TBS/T) containing 5% (*w*/*v*) nonfat milk powder for 1 hour at room temperature and then incubated with appropriate primary antibodies against TRAIL, IDO1, phosphor-STAT1, phosphor-STAT2, phosphor-JNK, phosphor-MAPK, or beta-actin (TRAIL from RD, IDO1 from CST, and others from Arigo) overnight at 4°C. Specific bound primary antibodies were detected by peroxidase-coupled secondary antibodies and enhanced chemiluminescence signaling kit (CST, Beverly, MA).

### 2.5. Cell Viability Assay

AFMSCs (5 × 10^4^, 1 × 10^5^, or 2 × 10^5^/ml) were plated in 96- or 6-well plates in DMEM complete media and incubated for 12 hours. For preactivation, AFMSCs were incubated with IFN-*β*, IFN-*γ*, or IFN-*β* plus IFN-*γ* (20 ng/ml each) for 12 hours, respectively. After thorough washing to remove IFNs, H460 or H460 hrGFP cells were added into the AFMSC-containing wells. After 36 hours of coculture in RPMI 1640 complete medium, the amount of LDH released into media was determined by a cytotoxicity LDH assay kit (Dojindo, Kumamoto, Japan) and the cell viability was measured using a Cell Counting Kit-8 (Dojindo, Kumamoto, Japan). For direct visualization of viable H460 hrGFP cells, the coculture cells were recorded and counted via Cell Imaging Multi-Mode Reader (BioTek, Winooski, VT) after washing twice in PBS and staining with Hoechst 33342 (2 *μ*g/ml, Sigma-Aldrich, St. Louis, MO). The role of TRAIL in the antitumor effects of IFN-primed AFMSCs was explored as in our previous report with some modifications [13]. Briefly, a neutralizing anti-TRAIL antibody (20 *μ*g/ml, R&D Systems, Minneapolis, MN) was added into the media at the beginning of coculture. PZ0304 (15 *μ*M, Sigma-Aldrich, St. Louis, MO), an inhibitor of Jak1, which is necessary to mediate signal transduction triggered by IFN-*β* and IFN-*γ*, and 1-MT (0.5 mM, Sigma-Aldrich, St. Louis, MO), an IDO1 inhibitor, were added into the media during priming of AFMSCs with IFN-*β* plus IFN-*γ*, respectively, in order to inhibit the expression of TRAIL in AFMSCs. All experiments were performed in triplicates and repeated at least three times.

### 2.6. Apoptosis and Caspase-3 Activation Assays

AFMSCs (1.5 × 10^5^/ml) were plated in 6-well plates in DMEM complete media and incubated for 12 hours. For preactivation, AFMSCs were incubated with IFN-*β*, IFN-*γ*, or IFN-*β* plus IFN-*γ* (20 ng/ml each) for 12 hours, respectively. After thorough washing to remove IFNs, H460 or H460 hrGFP cells (1.5 × 10^5^/ml) were added into the AFMSC-containing wells. After 24 and 36 hours of coculture, the mixtures of AFMSCs and H460 cells were collected, suspended in PBS, and incubated at 4°C with CD90-APC (BD Pharmingen, Palo Alto, CA) for 45 minutes, washed with PBS by centrifugation, incubated at RT with Annexin V-FITC and propidium iodide (PI) sequentially according to the manufacturer's instructions (SunGene, Tianjin, China), and analyzed by flow cytometry (Beckman Coulter, Brea, CA). In addition, the presence of activated caspase-3 in H460 hrGFP cells was measured using an anti-active caspase-3 pAb (Promega, Madison, WI) after 36 hours of coculture. All experiments were performed in triplicates and repeated at least three times.

### 2.7. Preparation of Apoptotic H460 Component-Treated AFMSCs

H460 cells were preplated overnight in RPMI 1640 complete media and then treated with 10 ng/ml rhTRAIL (Novoprotein, Shanghai, China). After being incubated for 10 hours at 37°C, floating cells were collected by centrifugation at 2000 rpm for 3 minutes. The media derived from apoptotic cells were first collected, and then the pellet was resuspended in PBS. Lastly, the media and apoptotic cells were added into AFMSC-containing wells, respectively. In addition, the apoptotic H460 cells were resuspended in 0.2 ml PBS containing either 20 *μ*g of RNase or 30 units of DNase (Sigma-Aldrich, St. Louis, MO) and incubated for 2 hours at 37°C. The cells were washed by centrifugation and resuspended in PBS before being added into AFMSC-containing wells.

### 2.8. Animal Studies

BALB/c and NOD-SCID mice were purchased from the experimental animal center of Guangdong Province and the Vital River Lab Animal Technology Co. Ltd. (Beijing, China), respectively. All animal studies were approved by the Institutional Animal Care and Use Committee of Kingmed School of Laboratory Medicine of Guangzhou Medical University. H460 cells were suspended in 150 *μ*l of PBS and subcutaneously injected into the left flank area of the mice (BALB/c: 4 × 10^6^, NOD/SCID: 2 × 10^6^). At the same time, AFMSCs or IFN-primed AFMSCs (BALB/c: 2 × 10^6^, NOD/SCID: 1 × 10^6^ or 2 × 10^6^) were implanted, after being admixed with tumor cells or alone. Another group of mice that received IFN-*γ*-primed AFMSCs were given 1-MT orally with 1 mg/ml of drinking water for the entire course before one day of injection. Mice were examined twice a week. Tumor sizes were calculated using the following formula: volume = length × width^2^/2, where length represents the major axis (largest cross-sectional diameter) of the tumor while width represents the minor axis. After 30 days, mice were sacrificed, tumors were excised, and tumor weights were compared.

### 2.9. Statistical Analysis

Data are expressed as means ± standard deviations (SDs). All results represent data collected from at least 3 independent experiments. Statistical analyses were conducted using one-way analysis of variance (ANOVA). A *P* value <0.05 was considered statistically significant.

## 3. Results

### 3.1. Characterization of AFMSCs

**Figure 1:**
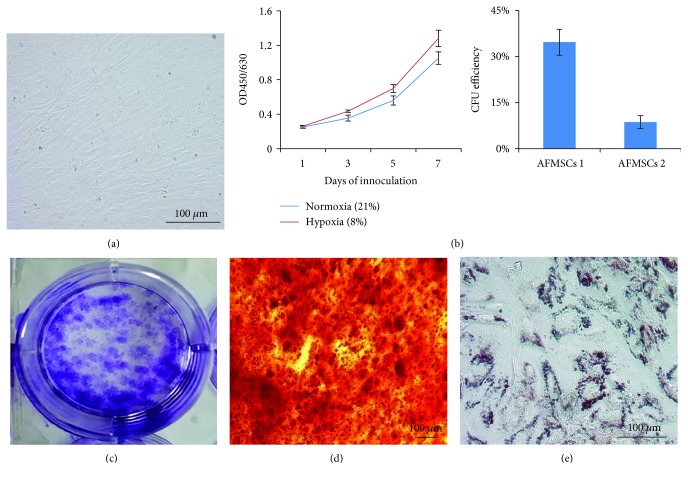
Characteristics of isolated human amniotic fluid-derived mesenchymal stem cells (AFMSCs). (a) Phase-contrast microscopy of spindle-shaped AFMSCs at passage 6. (b) Proliferation ability of AFMSCs under hypoxia and normoxia conditions was determined by CCK-8 counting. (c) Colony count in petri dish and colony formation efficiency in bar graph. (d) Alizarin Red S staining of osteogenic-differentiated AFMSCs. (e) Oil red O staining of adipogenic-differentiated AFMSCs. Scale bars 100 *μ*m (*n* = 3).

AFMSCs isolated from human amniotic fluid exhibited uniform spindle-like morphology, which was similar to that of bone marrow-derived MSCs (Figure 1(a)). The AFMSC proliferation was significantly faster under hypoxia than under normoxia (Figure 1(b)). The colony-forming unit capacity of two different original AFMSCs was determined, and their CFU efficiency was 34.6% and 8.7%, respectively (Figure 1(c)). These cells had multidifferentiation capability and could be induced into osteoblasts and adipocytes (Figures 1(d) and 1(e)). Flow cytometry analysis showed that CD44, CD73, CD90, CD105, and HLA-I were highly expressed in these cells, but HLA-II, CD34, and CD45 were not (Figure S1). The classic G-banded karyotyping was done on various passages (P1, 16, and 30) of ex vivo expanded AFMSCs. The results showed that there were no chromosomal abnormalities (Figure S2).

### 3.2. Distinct Effects of IFN-*β* and IFN-*γ* on Antitumor Activity of AFMSCs *In Vivo*

**Figure 2:**
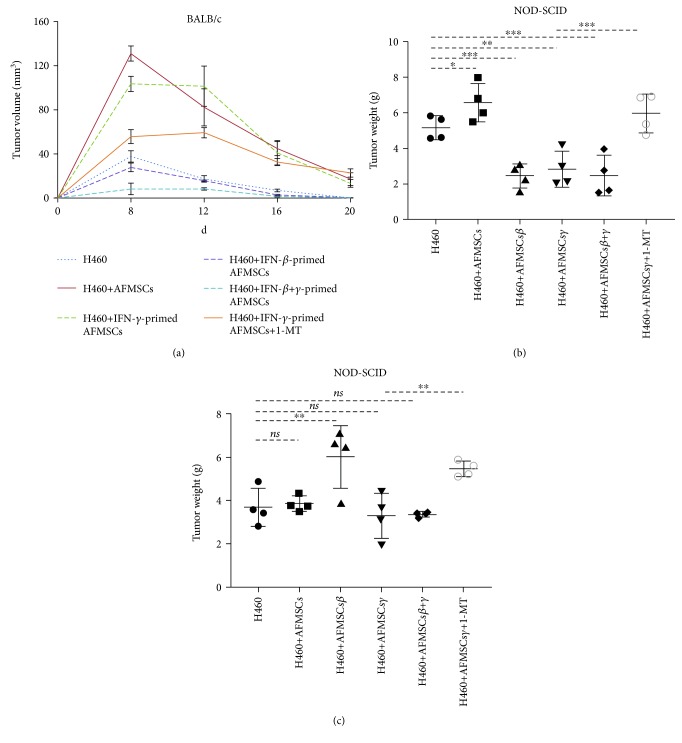
Comparison of antitumor activity of IFN-*β*-primed AFMSCs and IFN-*γ*-primed AFMSCs *in vivo*. (a) 4 × 10^6^ H460 cells with or without 2 × 10^6^ AFMSCs and IFN-*β*-primed, IFN-*γ*-primed, or IFN-*β*+IFN-*γ*-primed AFMSCs were injected into BALB/c mice subcutaneously. Tumor progression was monitored for 20 days, and tumor growth was expressed as the mean nodule size at various time points after tumor appearance. (b, c) 2 × 10^6^ H460 cells with or without 2 × 10^6^ (b) or 1 × 10^6^ (c) AFMSCs and IFN-*β*-primed, IFN-*γ*-primed, or IFN-*β*+IFN-*γ*-primed AFMSCs were injected into NOD/SCID mice subcutaneously. At 30 days after tumor inoculation, tumors were excised and weighed. In addition, another group of mice that received IFN-*γ*-primed AFMSCs were orally administered 1-MT at a dose of 1 mg/ml in drinking water before one day of injection. All values represent means ± SDs. Experiments were repeated 2 to 3 times. ns: not significant (^∗^ *P* < 0.05, ^∗∗^ *P* < 0.01, and ^∗∗∗^ *P* < 0.001, *n* = 4 or 5).

Previous studies have shown that IFN-*β*- or IFN-*γ*-modified MSCs could effectively inhibit the growth of tumor cells. To further elucidate the relevant mechanism, a xenograft H460 tumor model was constructed in immunocompetent and immunocompromised mice with or without various IFN-primed AFMSCs. First, H460 cells were subcutaneously injected into BALB/c mice. As shown in Figure 2(a), tumor mass approximately 4 mm in diameter was observed after eight days of inoculation and then gradually disappeared. AFMSCs or IFN-*γ*-primed AFMSCs dramatically promoted the earlier onset and delayed the disappearance of tumor mass without changing the kinetics of tumor growth. In contrast, IFN-*β*- or IFN-*β*+IFN-*γ*-primed AFMSCs delayed the onset and facilitated the disappearance of tumor mass. 1-MT, the inhibitor of immunosuppressive molecule IDO1, partially reduced the tumor-promoting activity of IFN-*γ*-primed AFMSCs.

Compared to the H460 group and H460 plus AFMSC group, IFN-*β*-, IFN-*γ*-, or IFN-*β*+IFN-*γ*-primed AFMSCs all inhibited the growth of tumor cells after being mixed with H460 tumor cells and injected into NOD/SCID mice (Figure 2(b)). In addition, the antitumor activity of IFN-primed AFMSCs was related to the ratio of tumor cells and AFMSCs in number. Moreover, 1-MT surprisingly decreased the antitumor activity of IFN-*γ*-primed AFMSCs (Figures 2(b) and 2(c)).

Taken together, these data demonstrated that AFMSCs or IFN-*γ*-primed AFMSCs could promote tumor growth by inducing host immune tolerance *in vivo*. On the other hand, IFN-*γ*-primed AFMSCs could also inhibit tumor growth by utilizing nonimmune strategy. Moreover, IFN-*β* could reverse the tumor-promoting properties of AFMSCs primed with or without IFN-*γ* and enable AFMSCs to directly inhibit the growth of tumor cells.

### 3.3. Expression of TRAIL and IDO1 in AFMSCs Regulated by IFN-*β* and IFN-*γ*

**Figure 3:**
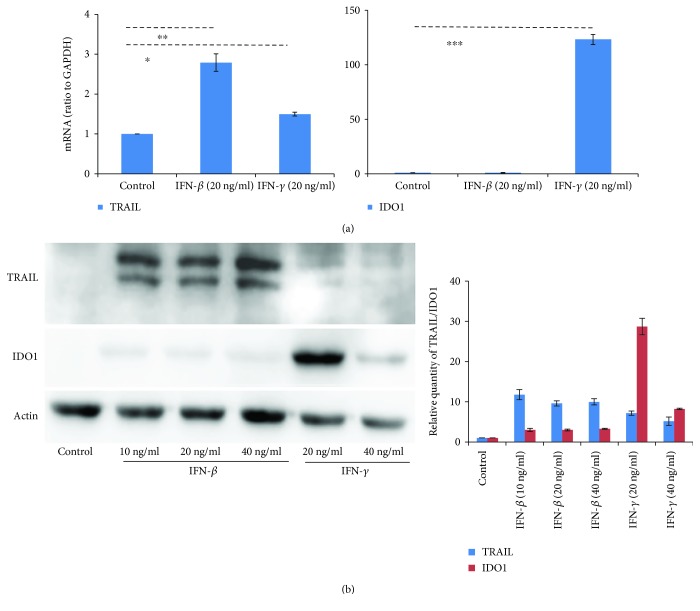
The expression of TRAIL in AFMSCs was initiated by both IFN-*β* and IFN-*γ*, but only IFN-*γ* initiated the expression of IDO1 in AFMSCs. (a) AFMSCs were primed for 12 hours with IFN-*β* or IFN-*γ* (20 ng/ml each). TRAIL and IDO1 mRNA were measured by real-time PCR, and GAPDH was used as an internal control. The expression levels were normalized to the level of GAPDH mRNA (defined as an arbitrary unit). (b) AFMSCs were primed with IFN-*β* or IFN-*γ* at specified concentrations for 12 hours. The protein levels of TRAIL and IDO1 were determined by western blotting analysis. Results were representative of three independent experiments (^∗^ *P* < 0.05, ^∗∗^ *P* < 0.01 and ^∗∗∗^ *P* < 0.001, *n* = 3).

Since TRAIL might participate in the antitumor activity of IFN-primed MSCs and IDO1 is the key effector molecule of MSC-mediated immunosuppression [13, 15], we then examined whether AFMSCs increased the expression of TRAIL and IDO1 after exposure to IFNs. As shown by RT-PCR analysis (Figure 3(a)), the relative levels of TRAIL mRNA in AFMSCs primed with the same concentration (20 ng/ml) of IFN-*β* and IFN-*γ* was upregulated by almost three and one and half times more than the control, respectively. In contrast, IFN-*γ*-primed AFMSCs expressed very high levels of IDO1 (over one hundred times more than the control group) while IFN-*β* had a minimal effect on the expression of IDO1 in AFMSCs. To further confirm the expression intensity of TRAIL and IDO1 in AFMSCs, we performed western blot on whole cell extracts from AFMSCs with or without IFN priming. We found that IFN-*β* and IFN-*γ* both induced a significant expression of TRAIL in AFMSCs, whereas only IFN-*γ* induced a significant expression of IDO1 in AFMSCs (Figure 3(b) and Figure S3).

**Figure 4:**
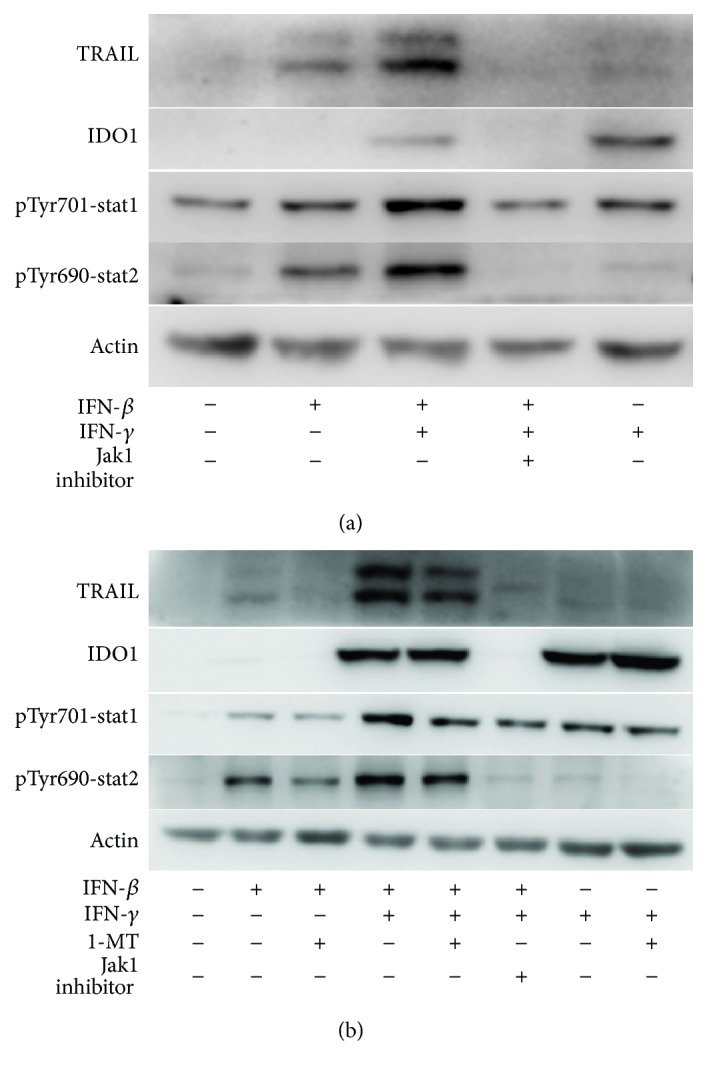
IFN-*β* interacted with IFN-*γ* to promote TRAIL expression and counteracted IDO1 expression initiated by IFN-*γ* in AFMSCs by regulating the phosphorylation of STAT1/2. 1-MT downregulated TRAIL expression in IFN-*β*- and IFN-*γ*-primed AFMSCs by inhibiting the phosphorylation of STAT1/2, but upregulated the IDO1 expression in IFN-*γ*-primed AFMSCs by intensifying the phosphorylation of JNK/MAPK38 (Supplemental Figure S3). (a) AFMSCs were primed for 12 hours with the indicated combinations of cytokines (20 ng/ml each) with or without Jak1 inhibitor (15 *μ*M). The protein levels of TRAIL, IDO1, and phosphorylated STAT1/2 were determined by western blotting analysis. (b) AFMSCs were treated as described in (a), in addition to setting a 1-MT (0.5 mM) combination. Results were representative of three independent experiments.

Type I and type II IFNs have been shown to share overlapping signaling pathways, such as the canonical JAK-STAT1/2 to initiate target gene expression [1]. However, our results indicated that the expression of TRAIL and IDO1 in AFMSCs was regulated differently by IFN-*β* and IFN-*γ*. Therefore, we investigated the relationship between the phosphorylation of STAT1 and STAT2 and the expression of TRAIL and IDO1 in AFMSCs. As shown in Figure 4(a), IFN-*β* increased phosphorylated STAT1 and STAT2, but IFN-*γ* increased phosphorylated STAT1 only. To clarify whether the formation of phosphor-STAT2 affected the expression of IDO1, AFMSCs were primed simultaneously with IFN-*β* and IFN-*γ*. Compared with control, although phosphor-STAT1 was also increased dramatically, the presence of phosphor-STAT2 was significantly related to the decrease in IDO1. Instead, IFN-*β* and IFN-*γ* synergistically promoted the expression of TRAIL. The special inhibitor PZ0304 of Jak1, a common signaling molecule of IFN-*β* and IFN-*γ*, decreased the phosphorylation of STAT1 and STAT2 and thus reduced the expression of TRAIL and IDO1 in IFN-primed AFMSCs (Figure 4(a)).

### 3.4. 1-MT Reduced the Expression of TRAIL, but Enhanced the Expression of IDO1

Based on the results of the animal trial (Figure 2), we speculated that the IDO1 inhibitor 1-MT might interfere with the expression of TRAIL and IDO1 in IFN-primed AFMSCs except that 1-MT inhibited the enzymatic activity of IDO1. We first examined the effects of 1-MT on STAT1/2 phosphorylation in IFN-primed AFMSCs. As shown in Figure 4(b) and Figure S4, 1-MT blocked the formation of phosphorylated STAT1/2. The expression of TRAIL was then correspondingly reduced, but the expression of IDO1 was unexpectedly enhanced. In addition to the JAK-STAT1/2 pathways, other JAK-dependent signaling cascades are also activated by IFNs, including the mitogen-activated protein kinase p38 (MAPK38) and JNK [1, 16]. So we further investigated the effect of 1-MT on MAPK38 and JNK phosphorylation in IFN-primed AFMSCs. The results indicated that 1-MT could partially promote the expression of phosphorylated JNK and MAPK38 in IFN-*γ*-primed AFMSCs (Figure S5).

### 3.5. IFN-Primed AFMSCs Attenuated the Growth of H460 Cells

**Figure 5:**
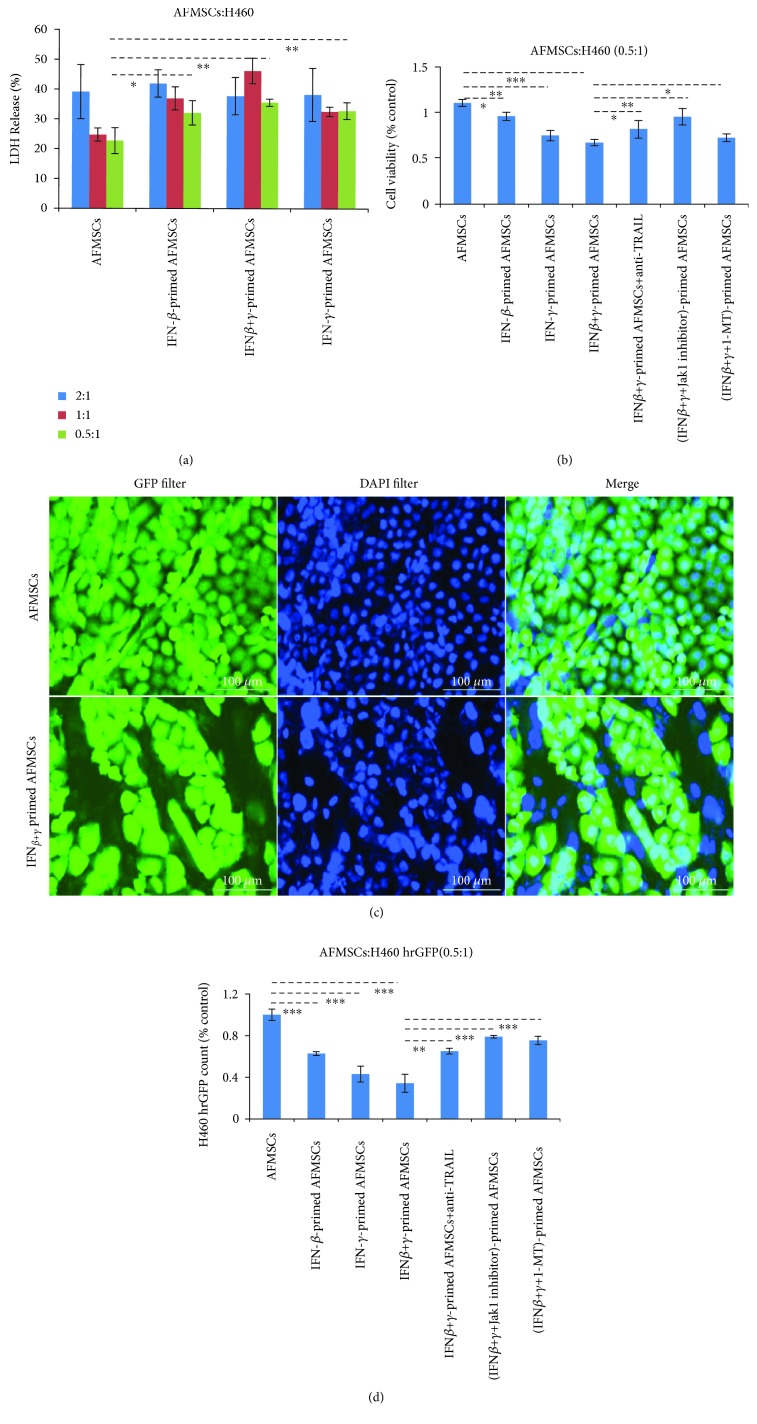
IFN-primed AFMSCs inhibited the proliferation of H460 cells ex vivo. (a) H460 cells (1 × 10^4^) were cocultured for 36 hours with AFMSCs or IFN-primed AFMSCs at different target-to-effector cell ratios. The effects of IFN-primed AFMSCs on LDH releases were determined. (b) AFMSCs were exposed to the indicated combinations of cytokines (20 ng/ml each) with or without Jak1 inhibitor (15 *μ*M)/1-MT (0.5 mM) for 12 hours, then cocultured with double the number of H460 cells in the presence or absence of anti-TRAIL antibody (20 *μ*g/ml) for 36 hours. The cell viability of each group was determined by CCK-8 counting. (c, d) The coculture experiment was prepared as described in (b) using H460 hrGFP cells instead of H460 cells. The representative photographs under a fluorescence microscope are shown (c), and the viability of H460 cells was further confirmed using Cell Imaging Multi-Mode Reader (d). Scale bars 100 *μ*m. The results in (b, d) were shown as a percentage of the value against AFMSC control. The results were representative of three independent experiments (^∗^ *P* < 0.05, ^∗∗^ *P* < 0.01, and ^∗∗∗^ *P* < 0.001, *n* = 3).

First, the cytotoxic effects of IFN-*β*- and IFN-*γ*-primed AFMSCs on H460 were determined and compared. As shown in Figure 5(a), the IFN-primed AFMSCs resulted in cell death in a dose-dependent manner with LDH release. The combination of CCK-8 assay and H460 hrGFP cell count was then used to allow a clear distinction between target and effector cells and to accurately estimate the viability of target cells. As shown in Figures 5(b) and 5(d), IFN-primed AFMSCs effectively attenuated H460 cell viability compared to control (*P* < 0.05). IFN-*β* and IFN-*γ* had synergistic effects on the antitumor proliferation of AFMSCs. We observed that the cytotoxic effects were partially blocked by special inhibitors of Jak1 and IDO1, respectively. The anti-TRAIL antibody also effectively reversed the cytotoxic effects induced by IFN-primed AFMSCs. These results clearly indicated that IFN-primed AFMSCs inhibited tumor cell proliferation, and TRAIL was involved in this progression.

### 3.6. IFN-Primed AFMSCs Induced Apoptosis of H460

**Figure 6:**
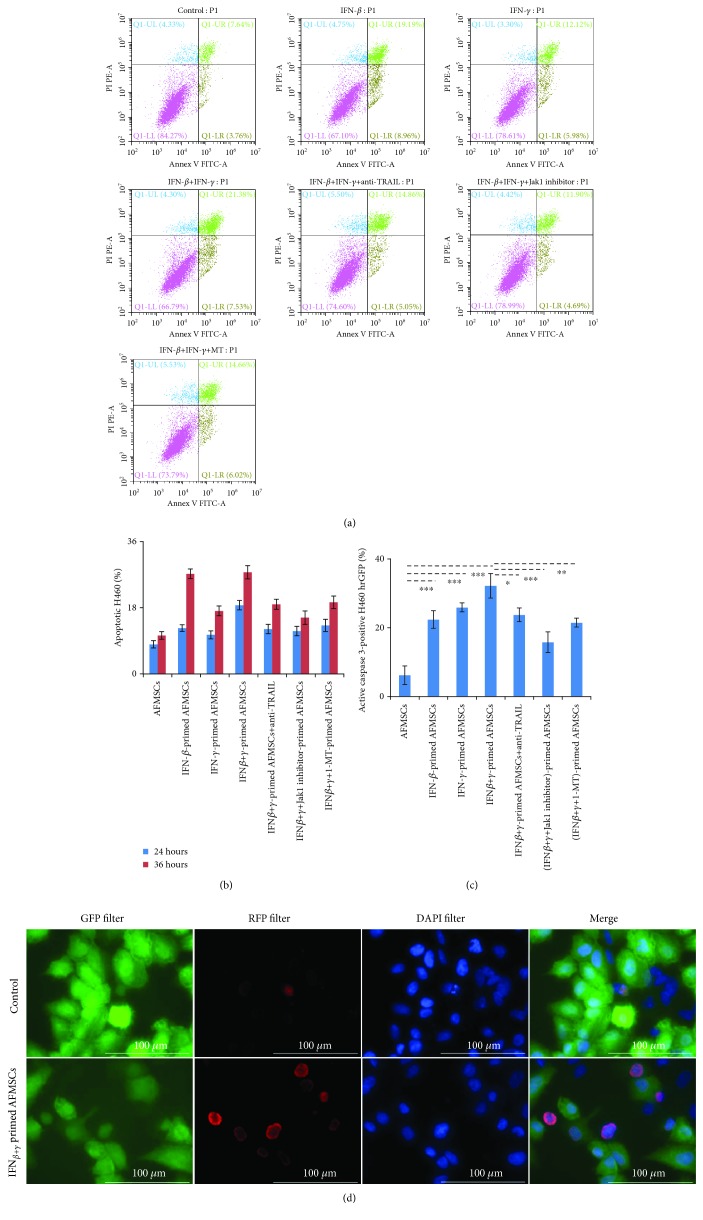
IFN-primed AFMSCs reduced the cell viability of H460 by the TRAIL-mediated apoptosis pathway. (a) AFMSCs were exposed to the indicated combinations of cytokines (20 ng/ml each) with or without Jak1 inhibitor (15 *μ*M)/1-MT (0.5 mM) for 12 hours, then cocultured with the same number of H460 cells in the presence or absence of anti-TRAIL antibody (20 *μ*g/ml) for 36 hours. Cells were collected and labeled with anti-CD90 antibody to distinguish H460 from AFMSCs and then stained with Annexin V-FITC & PI. The apoptosis of H460 cells was analyzed. (b) The percentage of apoptotic H460 at 24 hours and 36 hours was compared. (c, d) The samples in coculture experiments were prepared as described in (a) using H460 hrGFP cells instead of H460 cells. The activated caspase-3 within H460 hrGFP cells was detected by immunofluorescence staining. The proportions of activated caspase-3-positive H460 hrGFP cells were quantified (c), and the representative photographs in fluorescence microscope are shown (d). Scale bars 100 *μ*m. The results were representative of three independent experiments (^∗^ *P* < 0.05, ^∗∗^ *P* < 0.01, and ^∗∗∗^ *P* < 0.001, *n* = 3).

To determine whether the cytotoxicity of IFN-primed AFMSCs is mainly mediated by apoptosis or not, we conducted flow cytometric analysis and caspase-3 activation assay. First, AFMSCs and H460 cells could be distinguished by the anti-CD90 antibody, since the CD90 antigen was expressed on the surface of AFMSCs, but not on H460 (Figure S6). As shown in Figures 6(a) and 6(b) and Figure S7, IFN-primed AFMSC groups showed an increase in the number of apoptotic tumor cells in a time-dependent manner compared with the control group. After 36 hours of coculture, the corresponding percentages of apoptotic H460 cells in control, IFN-*β*, IFN-*γ*, and IFN-*β*+IFN-*γ*-primed AFMSC groups were 11.4%, 28.2%, 18.1%, and 28.9%, respectively. Moreover, the percentages of H460 hrGFP with active caspase-3 in IFN-primed groups were also higher than those in the untreated AFMSC group (Figures 6(c) and 6(d), *P* < 0.05). At the same time, when the expression or activity of TRAIL was blocked by special inhibitors or antibodies, the number of apoptotic target cells and the percentage of target cells with active caspase-3 would decrease accordingly. These results indicated that IFN-primed AFMSCs decreased cell viability through the TRAIL-mediated apoptosis pathway.

### 3.7. Apoptotic H460 Cells Initiated the Expression of TRAIL in AFMSCs

**Figure 7:**
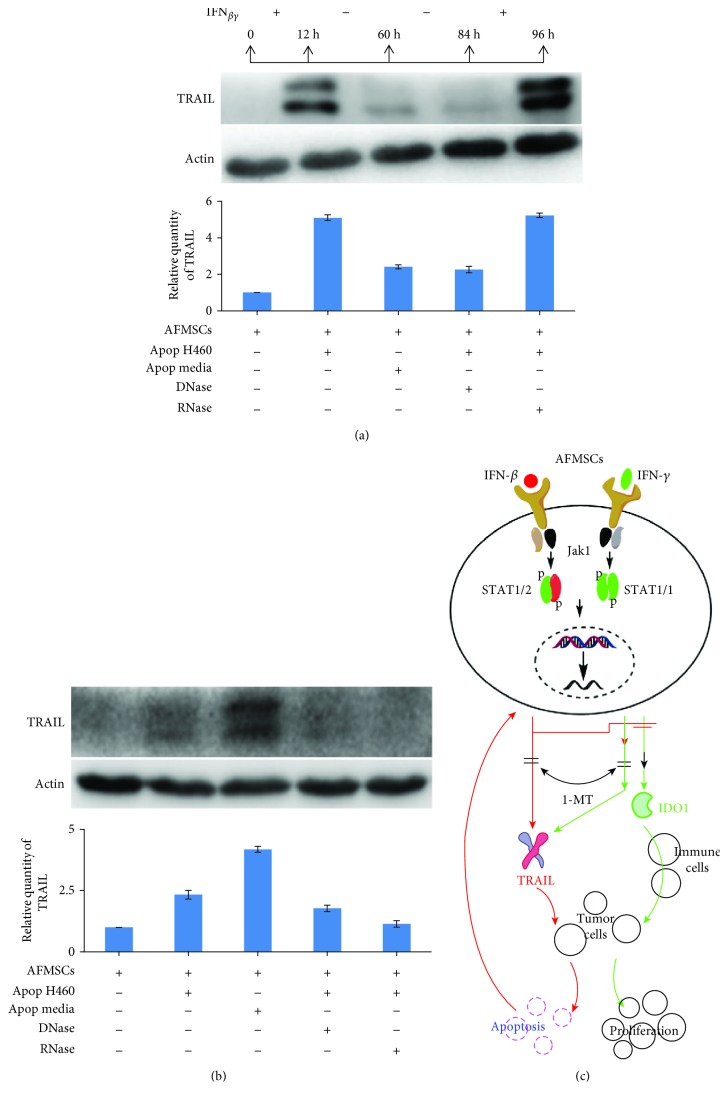
Apoptotic H460 cells initiated the expression of TRAIL in AFMSCs, which might provide a sustained antitumor effect. (a) The expression trait of TRAIL in IFN-primed AFMSCs was investigated by western blotting. IFN-*β*+IFN-*γ*-primed AFMSCs showed reduced expression of TRAIL after withdrawal of IFNs but continuously expressed TRAIL when primed again with IFNs. (b) AFMSCs were primed for 12 hours by apoptotic H460 cells, its culture media, or apoptotic H460 cells pretreated with DNase or RNase. The expression of TRAIL in AFMSCs was analyzed by western blotting. Results were representative of three independent experiments. (c) Graphic model describing the antitumor mechanism of IFN-primed AFMSCs and the effect of 1-MT on the antitumor activity of IFN-primed AFMSCs.

Based on the above results, it is clear that the sustained expression of TRAIL is the basic prerequisite to maintain the antitumor effects of AFMSCs. To explain why AFMSCs primed with IFNs just once had the antitumor effects *in vivo*, the expression pattern of TRAIL in AFMSCs was further investigated after AFMSCs were primed with IFN-*β* plus IFN-*γ* for 12 hours. As shown in Figure 7(a), the expression of TRAIL in AFMSCs gradually decreased with the withdrawal of the stimulus. However, once the stimulus reappeared, AFMSCs would restore the expression of TRAIL. We further explored whether the components derived from apoptotic H460 cells could also initiate the expression of TRAIL. As expected, media derived from apoptotic H460 cells and apoptotic H460 cells could induce AFMSCs to express TRAIL. In addition, pretreating apoptotic H460 cells with DNase or RNase decreased the expression of TRAIL in AFMSCs (Figure 7(b)).

## 4. Discussion

At present, MSCs could be separated and expanded from many tissues, such as bone marrow, muscle, and fat [6]. Here, our results showed that one subpopulation of amniocytes could proliferate quickly under hypoxic condition and embody classical characteristics of MSCs, including adhesion to plastic culture plates, typical spindle morphology, specific marker expression, and *in vitro* differentiation capacity. Furthermore, the biosafety of AFMSCs was further investigated by classic karyotyping in early- and late-passage cells. The results showed that extensive *in vitro* proliferation did not cause chromosomal abnormalities in AFMSCs. However, we were not entirely sure whether there exist point mutations or other subtle molecular events that might occur in cultured AFMSCs and make them prone to transformation. However, these expanded AFMSCs did not progress into tumor masses after being infused into immunocompromised mice (data not shown). Based on these results and relatively few ethical concerns, amniotic fluid can be considered as a source of MSCs for tissue engineering.

Owing to the tumor tropism properties, MSCs have been engineered to express various antitumor factors, such as IFN-*α*, TRAIL, and IL-12, and these genetically modified MSCs have shown dramatically therapeutic prospects [5, 17, 18]. But the immunosuppressive trait of MSCs might also bring the latent risk of promoting tumorigenesis [19]. On the other hand, the immunosuppressive capacity of MSCs was not always achieved [20, 21]. The source of MSCs and their local niche might determine their antitumor effects. For example, some studies have demonstrated that TNF-*α*-preactivated human bone marrow-derived MSCs suppressed the progression of lung tumors formed from breast cancer cells, while mouse bone marrow-derived MSCs preactivated with TNF-*α* promoted the tumorigenesis and metastasis of breast cancer [12, 22, 23]. Other researchers reported that human MSCs could be polarized into homogenously acting phenotypes called as MSC1 and MSC2 by Toll-like receptor signaling and further studies have shown that MSC1 attenuated tumor growth while MSC2 promoted tumor growth and metastasis [24, 25]. In addition, human adipose tissue-derived MSCs promoted melanoma cell progression, but had no effect on glioblastoma cells [26]. Therefore, how MSCs affect the development and progression of tumors deserves further study before their safe use in clinical practice. Our previous studies have demonstrated that human bone marrow-derived MSCs primed with IFN-*γ* could specifically inhibit the proliferation of lung cancer cells *in vitro* by directly inducing their apoptosis and their antitumor effects in partly immunocompromised mice were not as effective as *in vitro* [13]. It is reported that IFN-*α*-modified MSCs played an effective antitumor role *in vivo* mainly by immune-dependent ways [17]. Another report showed that the antitumor activity of IFN-*β* was not dependent on host immune response, but rather on the antiangiogenic effect mediated by nonhematopoietic cells [4]. These results prompted us to further explore the differences in the antitumor effects of IFN-I- and IFN-II-primed MSCs and their related mechanisms.

First, we found that IFN-*β*-primed AFMSCs inhibited the growth of H460 xenografts when they were coinoculated subcutaneously in both immunocompetent and immunocompromised mice. However, IFN-*γ*-primed AFMSCs showed contradictory effects in supporting tumor formation in immunocompetent hosts and inhibiting tumor formation in immunocompromised hosts. Interestingly, IDO1 inhibitor 1-MT could partially eliminate the tumor-promoting activity of IFN-*γ*-primed AFMSCs in immunocompetent hosts and completely reverse their antitumor activity in immunocompromised hosts. Compared with IFN-*β*-primed AFMSCs, IFN-*β* plus IFN-*γ*-primed AFMSCs had similar antitumor effects *in vivo*. The presence of MSCs has been reported to allow the proliferation of allogeneic tumor cells, which were otherwise rejected by immunocompetent recipients [19]. The IDO1-expressing humanized MSCs could inhibit the immune response and promote tumor growth in iNOS-deficient mice [27]. All these data suggested that IFN-*γ*-primed AFMSCs could properly eliminate the host's antitumor immunity and IFN-*β* counteracts this effect. IFN-*β*-primed AFMSCs worked in concert with the host's antitumor immunity to slow tumor growth. At the same time, a direct antitumor mechanism might simultaneously exist in IFN-primed AFMSCs.

It has been shown that inflammatory factor IFN-*γ* initiated the immunosuppressive effect of bone marrow-derived MSCs by inducing high expression of IDO1 [15]. In addition, IFN-*β* treatment increased TRAIL in serum of tumor patients and patients with the highest level of TRAIL showed a sustained tumor regression [28]. Our previous studies have also shown that IFN-*γ*-primed bone marrow-derived MSCs could express TRAIL and exhibit proapoptotic activity against tumor cells [13]. Therefore, we hypothesized that the antitumor and immunoregulatory effects of IFN-primed AFMSCs might be related to the expression of TRAIL and IDO1. Our results also confirmed that IFN-*β* and IFN-*γ* treatments could induce the expression of TRAIL in AFMSCs. Meanwhile, the expression of TRAIL regulated by IFN-*β* was more significant than that regulated by IFN-*γ*. There exist synergistic effects in regulating the expression of TRAIL between IFN-*β* and IFN-*γ*. Most interestingly, only IFN-*γ* induced IDO1 expression in AFMSCs, and IFN-*β* counteracted this effect of IFN-*γ*. This may be related to the formation of the STAT1-STAT2 heterodimer other than the formation of the STAT1-STAT1 homodimer [29]. Our results also confirmed that the phosphorylation of both STAT1 and STAT2 is more likely to occur in IFN-*β*- or IFN-*β*+IFN-*γ*-primed AFMSCs than in IFN-*γ*-primed AFMSCs, which might contribute to the formation of the STAT1-STAT2 heterodimer. These results explained why the antitumor effects of IFN-*β*- and IFN-*β* plus IFN-*γ*-primed AFMSCs on H460 cells were better than that of IFN-*γ*-primed AFMSCs ex vivo and *in vivo*. The simultaneous expression of IDO1 and TRAIL in IFN-*γ*-primed AFMSCs suggested a paradoxical role with IFN-*γ*-primed AFMSCs indirectly promoting tumor progression *in vivo* through immunosuppression and, on the other hand, directly inducing apoptosis in target cells, as demonstrated by coculture experiments *in vitro*. IDO1 inhibitor 1-MT synergistically promoted the expression of IDO1 and antagonistically blocked the expression of TRAIL in IFN-primed AFMSCs, which resulted in the elimination of direct cytotoxicity of IFN-*γ*-primed AFMSCs to tumor cells. These results further confirmed that 1-MT might promote rather than inhibit tumor progression by increasing IDO1 expression [30] and inhibiting TRAIL expression. Therefore, it is hard to say that the antitumor activity of IFN-*γ*-primed AFMSCs could be improved by combining with IDO1 inhibitor 1-MT.

We observed that IFN-primed AFMSCs could inhibit the proliferation of H460 cells by directly inducing TRAIL-mediated apoptosis and caspase-3 activation, but the expression of TRAIL in AFMSCs lasted only about 72 hours after withdrawal of the stimulus. Cell pellets and culture media derived from apoptotic tumor cells could also initiate the expression of TRAIL, which can be partially explained by RNA and DNA released from apoptotic cells because the treatment of apoptotic cells with RNase and DNase decreased their effectiveness of TRAIL expression in AFMSCs. Other researchers also reported that DNA and RNA from apoptotic tumor cells may increase the interactions of additional damage-associated molecular patterns (DAMPs) with hMSCs through different TLRs and enhance TRAIL expression [12, 31]. Therefore, this positive feedback response, which increased the expression of TRAIL, could provide the powerful effects of IFN-primed AFMSCs in suppressing tumor progression *in vivo*, even if the cells were engrafted only once in the tumors.

We also found more significant tumor growth in mice treated with AFMSCs compared to H460 alone. The reasons behind this phenomenon deserve further investigation. It may be that MSCs promote tumor development by triggering angiogenesis [13, 32]. However, the antitumor effect of IFN-primed AFMSCs could counteract the tumor-supportive capacity of AFMSCs and thus determine the efficiency of antitumor activity.

## 5. Conclusion

As summarized in Figure 7(c), our data further deepen the understanding of the importance and differences in the antitumor effects of type I interferon and type II interferon-primed MSCs, and the possible mechanisms of IFN-primed MSCs affecting tumor progression are also revealed. These results provide new molecular insights into the important roles of MSCs and interferon, which will contribute to the development of potential therapeutic approaches in the context of malignant diseases.

## Data Availability

The data used to support the findings of this study are available from the corresponding author upon request.

## Supplementary Materials

Supplementary Materials Figure S1: the cell-surface marker profile of AFMSCs. Figure S2: the normal G-banded karyotype of AFMSCs at passage 1 (a), 16 (b), and 30 (c). Figure S3: IFN-*γ* induced AFMSCs to express TRAIL and IDO1. Figure S4: 1-MT inhibited the phosphorylation of STAT1 (a) and STAT2 (b) in IFN-*β*- and IFN-*γ*-primed AFMSCs. Figure S5: 1-MT upregulated the expression of IDO1 in IFN-*γ*-primed AFMSCs by intensifying the phosphorylation of JNK/MAPK38. Figure S6: the expression of CD90 on the surface of AFMSCs and H460 cells was determined by flow cytometry. Figure S7: the apoptosis of H460 cells was analyzed by flow cytometry after being cocultured with different preparations of AFMSCs for 24 hours.
