# Supplementary material for: The Different Effects of IFN-β and IFN-γ on the Tumor-Suppressive Activity of Human Amniotic Fluid-Derived Mesenchymal Stem Cells
Source: Stem Cells Int. 2019 Apr 15;2019:4592701. doi: 10.1155/2019/4592701 (PMC6501177; doi:10.1155/2019/4592701)
Supplement: Supplementary Materials — Figure S1: the cell-surface marker profile of AFMSCs. Figure S2: the normal G-banded karyotype of AFMSCs at passage 1 (a), 16 (b), and 30 (c). Figure S3: IFN-γ induced AFMSCs to express TRAIL and IDO1. Figure S4: 1-MT inhibited the phosphorylation of STAT1 (a) and STAT2 (b) in IFN-β- and IFN-γ-primed AFMSCs. Figure S5: 1-MT upregulated the expression of IDO1 in IFN-γ-primed AFMSCs by intensifying the phosphorylation of JNK/MAPK38. Figure S6: the expression of CD90 on the surface of AFMSCs and H460 cells was determined by flow cytometry. Figure S7: the apoptosis of H460 cells was analyzed by flow cytometry after being cocultured with different preparations of AFMSCs for 24 hours. [file 4592701.f1.docx]

**
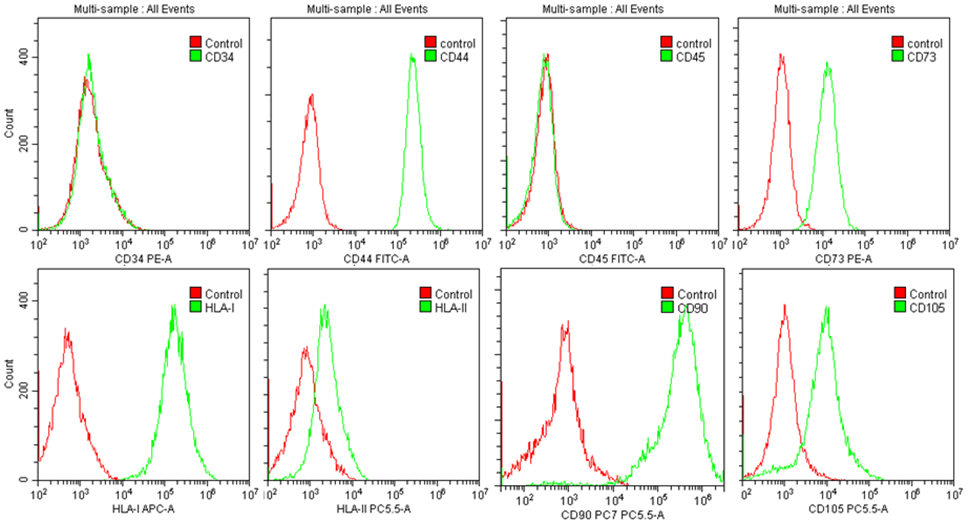
**

**Figure S1** The cell-surface marker profile of AFMSCs.

**
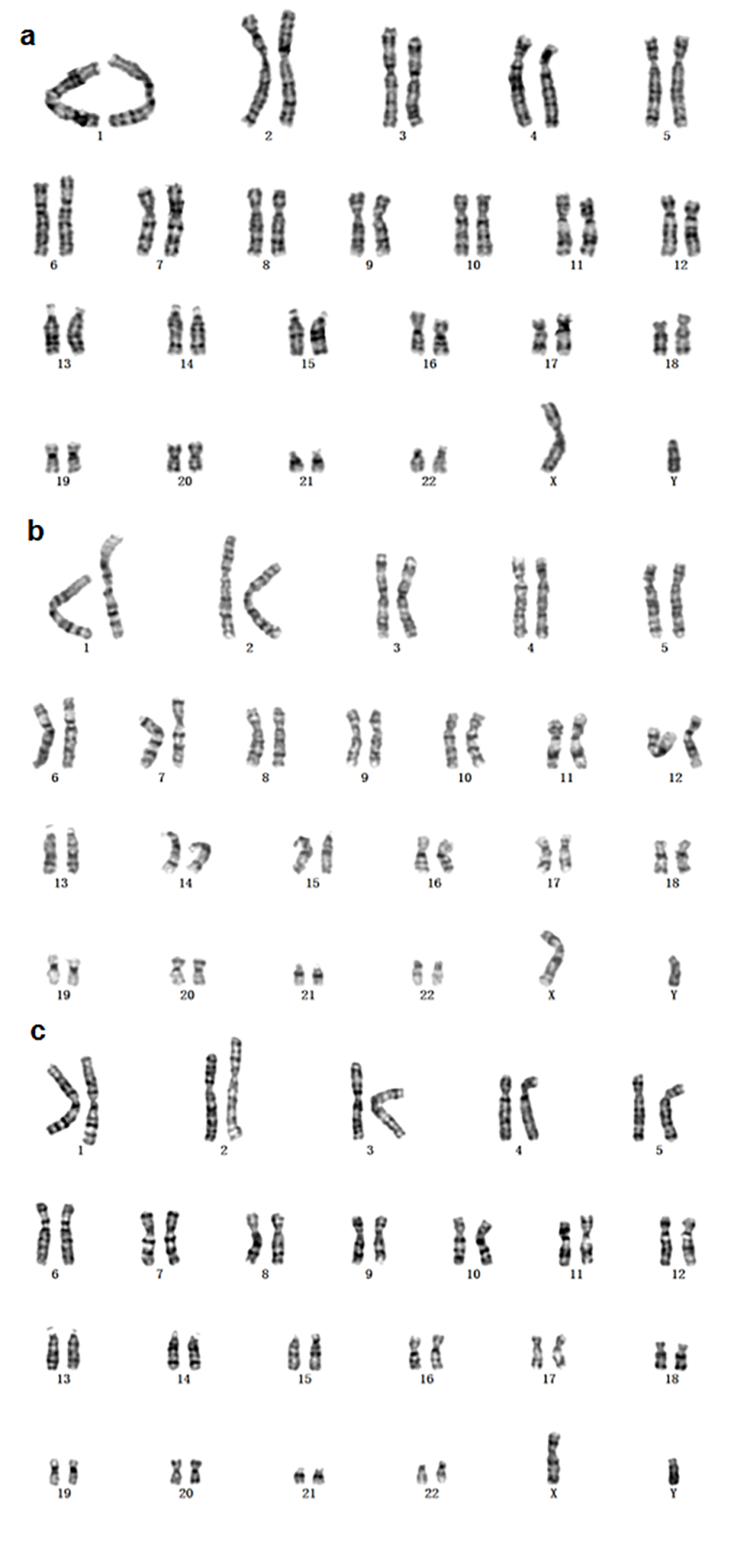
**

**Figure S2** The normal G-banded karyotype of AFMSCs at passage 1(a), 16 (b) and 30 (c).


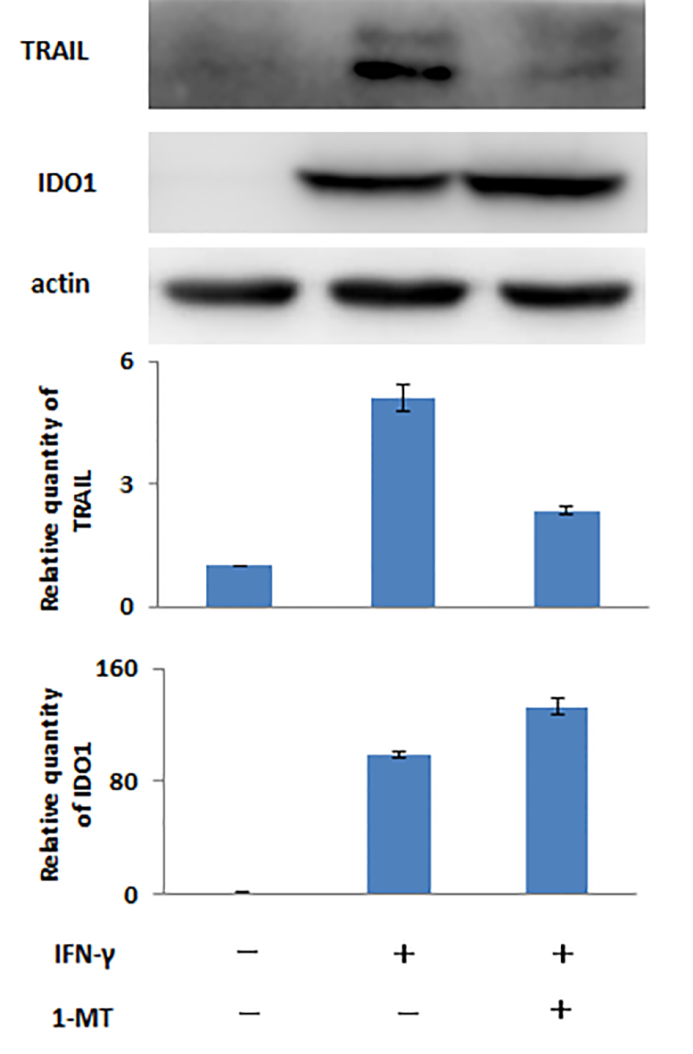


**Figure S3** IFN-γ induced AFMSCs to express TRAIL and IDO1. AFMSCs were primed with IFN-γ (20 ng/ml) for 12 hours. The protein levels of TRAIL and IDO1 were determined by western blotting analysis and quantified by densitometry analysis using Image Analysis Software. Results were representative of three independent experiments.


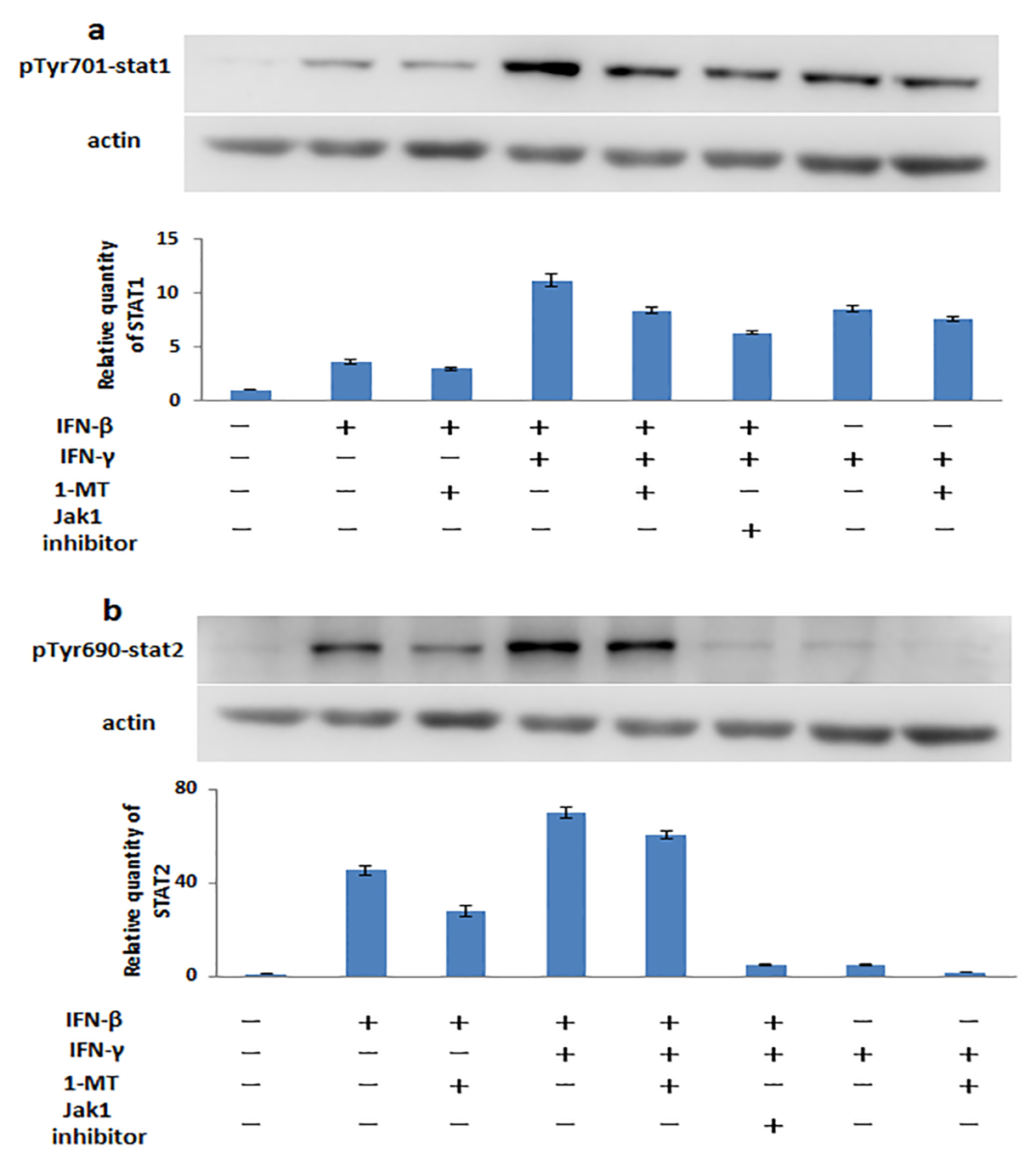


**Figure S4** 1-MT inhibited the phosphorylation of STAT1 (a) and STAT2 (b) in IFN-β and IFN-γ primed AFMSCs. AFMSCs were primed with the indicated combinations of cytokines (20 ng/ml each) with or without Jak1 inhibitor (15 µM) and 1-MT (0.5 mM) for 12 hours. The protein levels of phosphorylated STAT1/2 were determined by western blotting and quantified by densitometry analysis using Image Analysis Software. Results were representative of three independent experiments.


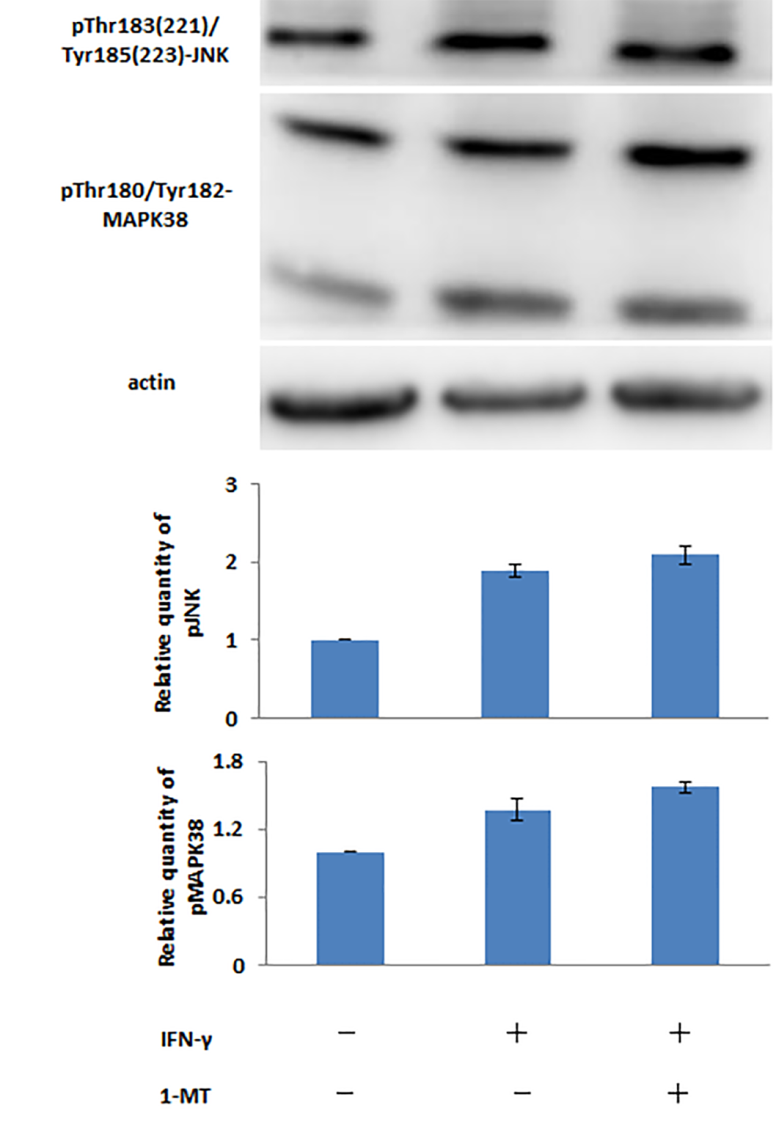


**Figure S5** 1-MT upregulated the expression of IDO1 in IFN-γ-primed AFMSCs by intensifying phosphorylation of JNK/MAPK38. AFMSCs were primed with IFN-γ (20 ng/ml) with or without 1-MT (0.5 mM) for 12 hours. The protein levels of phosphorylated JNK/MAPK38 were determined by western blotting analysis and quantified by densitometry analysis using Image Analysis Software. Results were representative of three independent experiments.


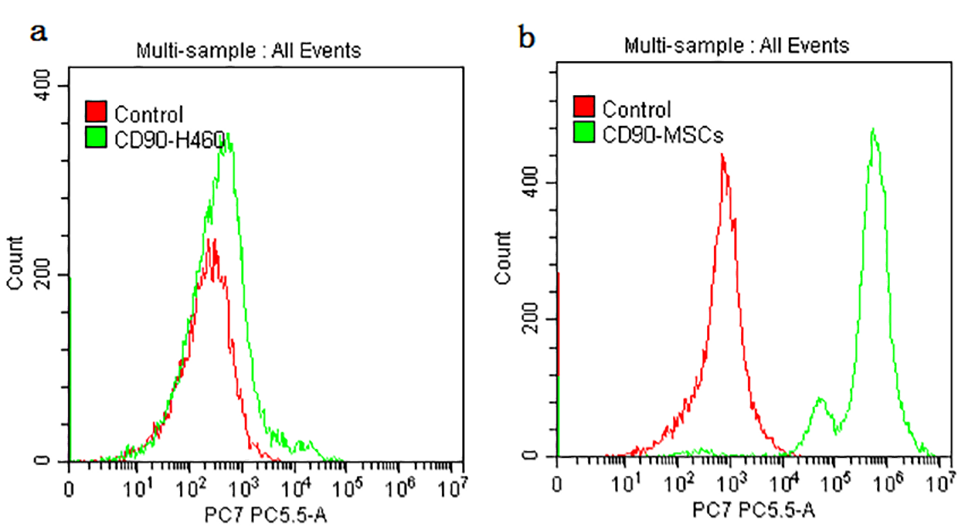


**Figure S6** The expression of CD90 on the surface of H460 (a) and AFMSCs (b) cells were determined by flow cytometry.


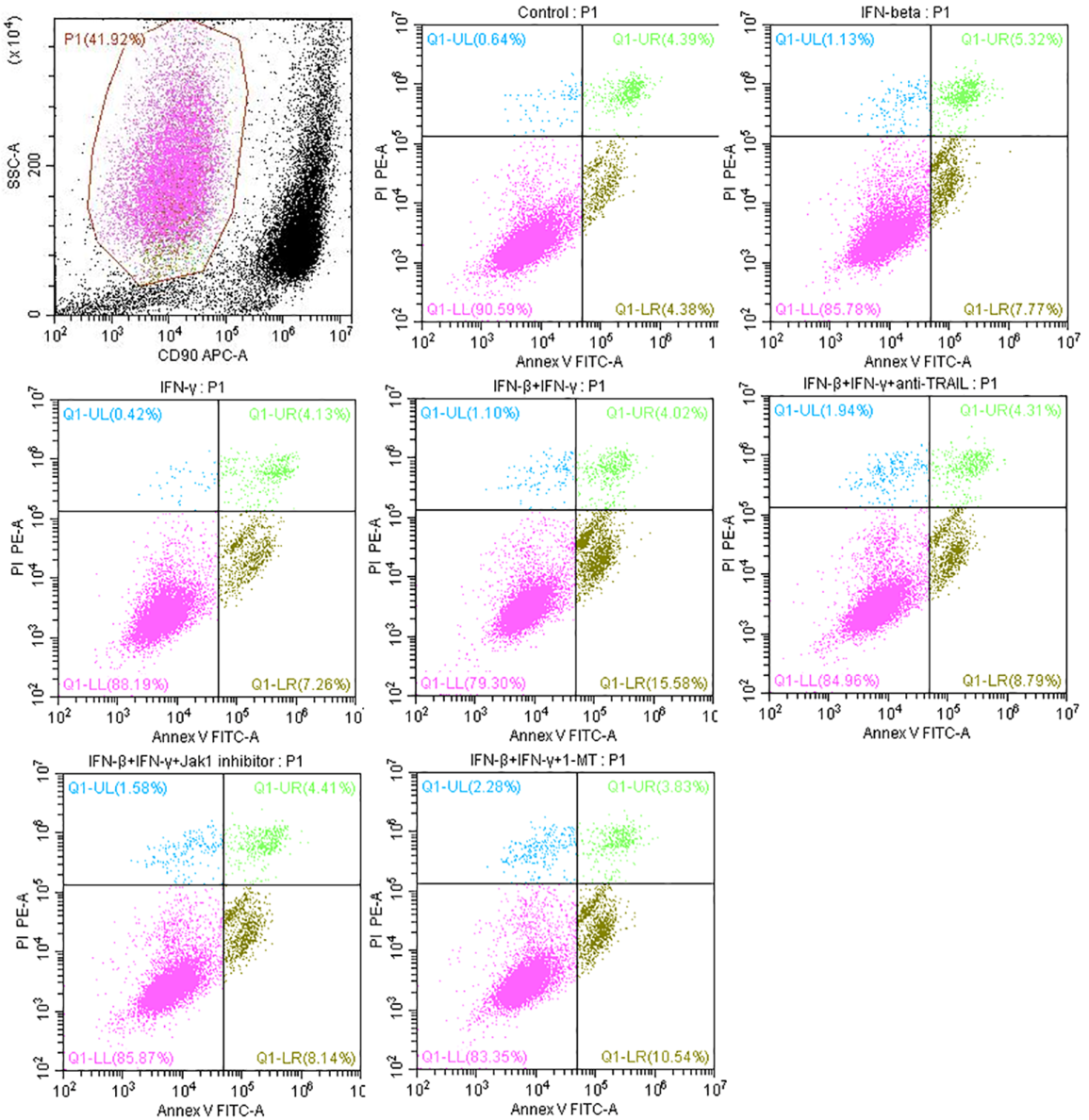


**Figure S7** The apoptosis of H460 cells was analyzed after cocultured with different preparations of AFMSCs for 24 hours. AFMSCs were exposed to the indicated combinations of cytokines (20 ng/ml each) with or without Jak1 inhibitor (15 µM)/ 1-MT (0.5 mM) for 12 hours, then cocultured with same number of H460 cells in the presence or absence of anti-TRAIL antibody (20 µg/ml) for 24 hours. Cells were collected and labeled with anti-CD90 antibody to distinguish H460 from AFMSCs, and then stained with Annexin V-FITC & PI. The results were representative of three independent experiments.
